# An approach to validate groundwater contamination risk in rural mountainous catchments: the role of lateral groundwater flows

**DOI:** 10.1016/j.mex.2018.11.002

**Published:** 2018-11-07

**Authors:** F.A.L. Pacheco, L.M.O. Martins, M. Quininha, A.S. Oliveira, L.F. Sanches Fernandes

**Affiliations:** a Department of Geology, University of Trás-os-Montes e Alto Douro, Ap. 1013, 5001-801 Vila Real, Portugal; b Chemistry Research Centre, Vila Real, Portugal; c Department of Civil Engineering, University of Trás-os-Montes and Alto Douro, Ap. 1013, 5001-801 Vila Real, Portugal; d Centre for the Research and Technology of Agro-Environment and Biological Science, Vila Real, Portugal

**Keywords:** An approach to validate groundwater contamination risk in rural mountainous catchments: the role of lateral groundwater flows, groundwater contamination risk, rural mountainous catchment, contaminant transport, travel time

## Abstract

Computer models dedicated to the validation of groundwater contamination risk in the rural environment, namely the risk of contamination by nitrate leachates from agriculture fertilizers, are frequently based on direct comparison of risky areas (e.g., cropland, pastures used for livestock production) and spatial distributions of contaminant (nitrate) plumes. These methods are fated to fail where lateral flows dominate in the landscape (mountainous catchments) displacing the nitrate plumes downhill and from the risky spots. In these cases, there is no connection between the spatial location of risky areas and nitrate plumes, unless the two locations can be linked by a contaminant transport model. The main purpose of this paper is therefore to introduce a method whereby spatio-temporal links can be demonstrated between risky areas (contaminant sources), actual nitrate plumes (contaminant sinks) and modeled nitrate distributions at specific groundwater travel times, thereby validating the risk assessment. The method assembles a couple of well known algorithms, namely the DRASTIC model [1,2] and the Processing Modflow software (https://www.simcore.com), but their combination as risk validation method is original and proved efficient in its initial application, the companion paper of Pacheco et al. [3].

## Highlights

• Existing risk validation models are based on the “spot matching” concept whereby validation is static and linked to the resemblance among spatial distribution of risky areas and regions with abnormally high nitrate concentrations;
• The current model is based on the “cross-profiling” concept whereby validation is dynamic and linked to the resemblance among nitrate profiles measured along catchment flow lines and modeled counterparts obtained for specific groundwater travel times.

## Graphical abstract

**Figure:**
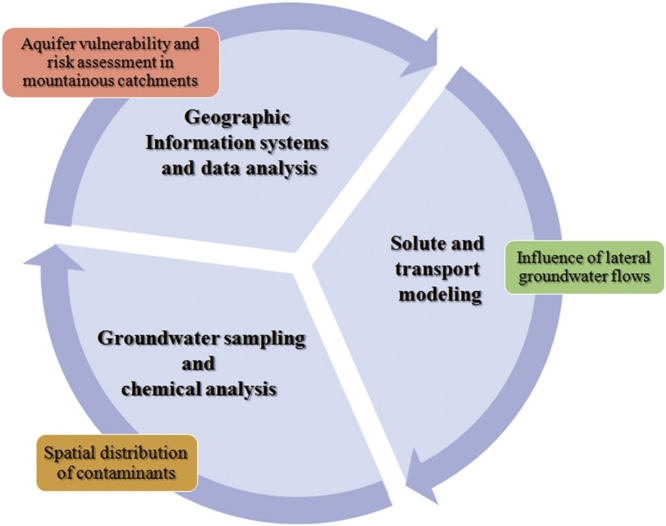

**Specifications Table**

**Table:** 

Subject area	*Environmental Science*
More specific subject area	*Hydrology* *Water resources*
Method name	*An approach to validate groundwater contamination risk in rural mountainous catchments: the role of lateral groundwater flows*
Name and reference of original method	*This method article is presented for the first time in the companion paper* [3]
Resource availability	*Links to data resources are provided in Table 1. Data used in* Pacheco et al. [3] *is provided as Supplementary Material*

## Method details and applicability

**Fig. 1:**
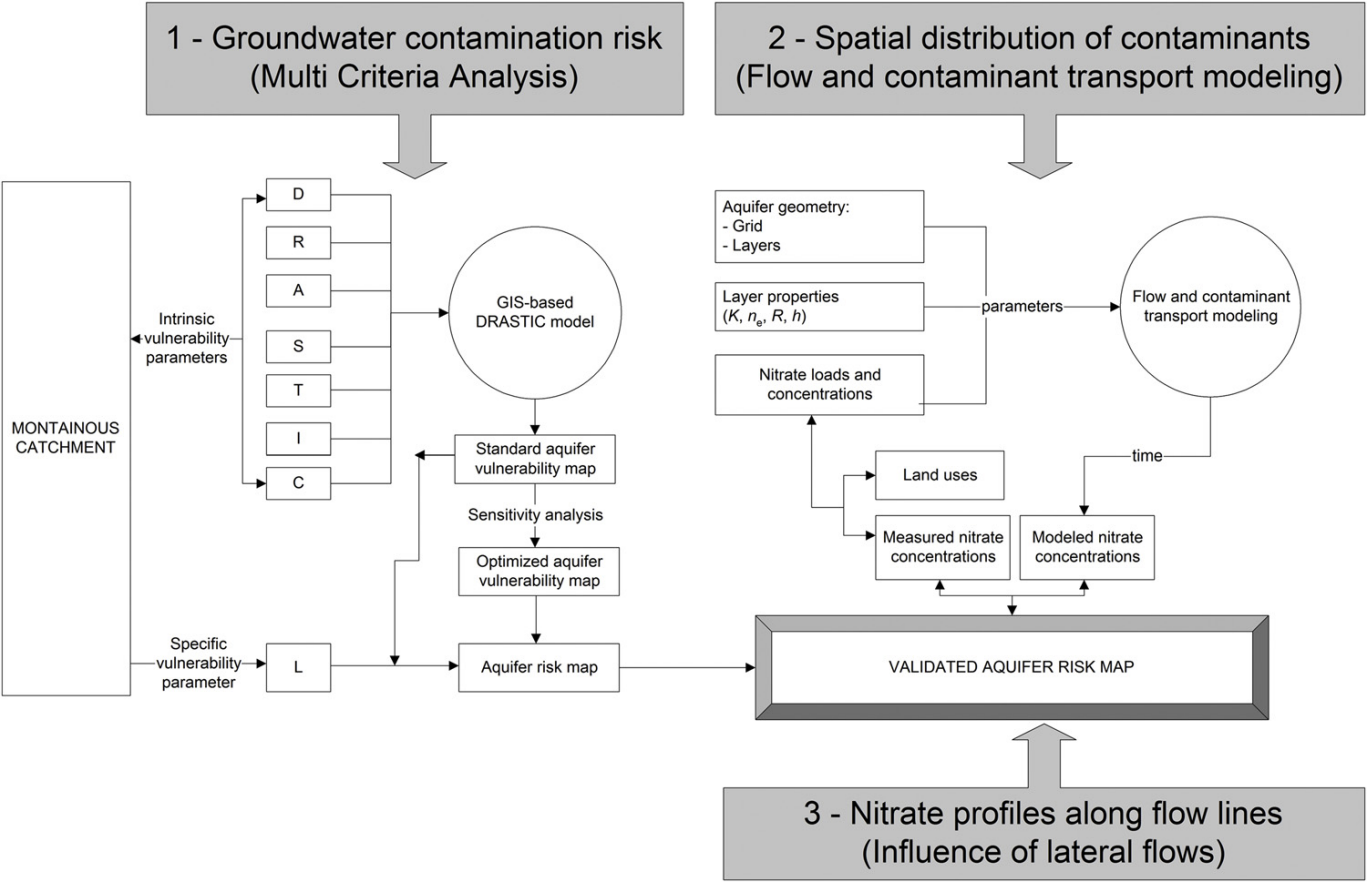
xxx.

The workflow to validate groundwater contamination risk in mountainous catchments is composed of three connected modules (Fig. 1): (1) the *risk assessment module*, which uses the DRASTIC model [1,2] to quantify aquifer intrinsic vulnerability, land use / land occupation data to quantify specific vulnerability [4], and an algebraic combination of intrinsic and specific vulnerability to quantify groundwater contamination risk; (2) the *groundwater flow-contaminant transport module*, which uses (*a*) stream flow models at catchment scale to estimate average aquifer properties (hydraulic conductivity and effective porosity) and groundwater travel time, (*b*) the Processing Modflow software (https://www.simcore.com) to model the spreading of nitrate loads injected at high risk spots; (3) *the validation module*, which compares measured and modeled nitrate concentrations along catchment profiles, with the main purpose of verifying if the measured concentrations can be related to the injection points via advective-dispersive transport during a specific time span. The three modules are described in detail in the next subsections. In all cases, they are complemented with the use of Geographic Information Systems (the ArcMap and ArcHydro computer packages of ESRI company), namely to perform necessary terrain modeling analyses, spatial interpolations, raster map computations, final thematic maps, among other geographic related tasks. The ArcMap software is widely used in environmental studies (e.g., [[5], [6], [7], [8], [9]]).

The risk maps produced with the present method can be included in management plans to help water authorities protecting local groundwater resources, as proposed in Valle et al. [10]. Other applications may include the embedding of vulnerability maps in hydrologic spatial decision support systems such as Mike Basin (e.g., [11,12]), to improve scenario analyses focused on groundwater protection. Besides application on water resources management and aquifer protection, the results from the present method can be coupled with outcomes from geochemical models, to improve the interpretation of groundwater composition in aquifers with substantial anthropogenic influence (e.g., [[13], [14], [15]]).

In mountainous watersheds where aquifer systems are primarily composed of carbonate rocks, the use of DRASTIC model in the risk assessment module may not be adequate. In these cases, it will have been preferable to describe intrinsic vulnerability using more specific methods, such as the EPIK [16], RESK [17], RISKE [18] or SINTACS [19], among others.

### Data

The sources of information and data required to implement the workflow are depicted in Table 1. This table refers to a specific application, namely the one presented in the companion paper where this method is applied for the first time [3], but it can be used as reference to any similar application.

**Table 1:** Sources of information and data required to implement the cross-profiling algorithm.. Symbol description (institution names in Portuguese): SNIRH – Sistema Nacional de Informação em Recursos Hídricos; DGT – Direção Geral do Território.

Code	Parameter	Unit	Information/data	Source	URL of internet website
*Risk assessment module (DRASTIC intrinsic vulnerability + land use-based specific vulnerability)*					
D	Depth to the water table	m	Hydraulic head data from 1261 dug wells located in the Morais Massif, northern Portugal	Pacheco, (2000)	
R	Recharge	mm·yr^–1^	Recharge data from 23 spring watersheds located around the Limãos, Assureira and Amedo sub-basins	Pacheco, (2000)	
A	Aquifer material	dimensionless	Spatial delineation and description of aquifer systems located in the Ancient Massif, published in the handbook of Almeida et al. (2000) entitled *The aquifer systems of Continental Portugal*	Water Institute, National information System on Water Resources	http://snirh.apambiente.pt/
S	Soil type	dimensionless	Spatial distribution of soil types	Agroconsultores and Coba (1991)	
T	Topography	%	Hillside slopes obtained from analysis of a Digital Elevation Model (DEM)	DGT	http://www.igeoe.pt
I	Impact of the vadose zone	dimensionless	Description of aquifer systems located in the Ancient Massif	Water Institute, National information System on Water Resources	http://snirh.apambiente.pt/
C	Hydraulic Conductivity	m·day^–1^	Transmissivity and thickness data from the 23 spring watersheds	Pacheco, (2000)	
L	Land Use	dimensionless	Specific vulnerability evaluation	Antonakos & Lambrakis, [4]	
*Groundwater flow and contaminant transport module (Processing Modflow)*					
*h*	Hydraulic head	m	Spatial distribution of hydraulic heads estimated from digital elevation (H) and depth to the water table (D) data	DGT; Pacheco, (2000)	http://www.igeoe.pt
*K*, *n*_e_	Hydraulic conductivity and effective porosity	m·day^–1^, dimensionless	Hydraulic conductivity and effective porosity of 23 spring watersheds calculated by the Brutsaert Method	Pacheco, (2000)	
*Q*	Spring discharge rate	m^-3^.s^-1^	Discharge of spring water measured on a monthly basis	Pacheco, (2000)	
NO_3_	Nitrate concentrations	mg·L^–1^	Nitrate concentration in the 23 spring watersheds, assessed at the spring site in April 1997, September 1997 and January 1998	Pacheco, (2000)	

The Supplementary Material composed of numeric and graphical data was assembled per module and sub-module and is provided in Excel format. Besides raw data used in the workflow (e.g., depths to water table measured in dug wells), these Excel worksheets contain the implementation of most equations of modules (1), (2a) and (3) presented below.

### Risk assessment module

The estimation of aquifer vulnerability is based on the DRASTIC model [1,2]. This method relates aquifer vulnerability to seven features that form the DRASTIC acronym: *D* - Depth to the water table, *R* - Recharge, *A* - Aquifer material, *S* - Soil type, *T* - Topography, *I* - Impact of the vadose zone and *C* - Hydraulic conductivity. Some features are numerical (e.g., *D*, *R*) while others are categorical (e.g., *A*, *S*). Regardless the type, features are evaluated within the studied area and then converted into a common dimensionless scale (ratings) to become consistently aggregable and mutually comparable. The ratings vary from 1 (lowest vulnerability) to 10 (highest vulnerability). The weighted sum (aggregation) of these ratings forms the DRASTIC index (*V*).

(1) $$V=\left(D_{w}\times D_{r}\right)+\left(R_{w}\times R_{r}\right)+\left(A_{w}\times A_{r}\right)+\left(S_{w}\times S_{r}\right)+\left(T_{w}\times T_{r}\right)+\;\left(I_{w}\times I_{r}\right)+\left(C_{w}\times C_{r}\right)$$

where subscript *r* points to the feature rating and subscript *w* to the feature weight. Factor weights were originally set up by an Environmental Protection Agency (EPA) committee using a Delphi (consensus) approach. The weights were assumed constant, with values *D_w_* = 5, *R_w_* = 4, *A_w_* = 3, *S_w_* = 2, *T_w_* = 1, *I_w_* = 5, and *C_w_* = 3.

The consensus on feature weights resulted from the understanding of real-world conditions at various well-studied regions. In many places, however, the importance given to the features is not understood because weights do not reproduce local conditions [20,21]. For that reason, application of DRASTIC model is frequently succeeded by a stage called sensitivity analysis, whereby original weights are replaced by optimized weights calculated by an objective function. The most common approaches to sensitivity analysis are the single-parameter [22] and the map removal [23] methods. The single-parameter method has the purpose to check the spatial significance of DRASTIC weights. While the original weights were given higher or lower values ignoring the spatial distribution of corresponding ratings, Napolitano and Fabbri [22] argue that effective weights should result from an assessment and judgment of *w*_j_*R*_j_ products across the entire studied region. Firstly, the authors identified unique condition subareas, which are parcels with specific combinations of weights (*w*) and ratings (*R*). Then, the effective or modified weight (*W*) of feature *j* was calculated by:

(2) $$W_{j}=\frac{\sum_{i=1}^{n}\frac{{w_{j}R}_{ij}}{V_{i}}}{n}$$

where *V*_i_ is the vulnerability index of parcel *i* and *n* is the number of parcels. The map removal method evaluates whether or not is necessary to use all the parameters in the assessment of DRASTIC vulnerability. Firstly, Lodwick et al. [23] defined the “unperturbed” and “perturbed” vulnerability indices. The DRASTIC index calculated by Equation 1 is the unperturbed index (*V*) based on *N* features, while the values computed using a lower number of features (*n*) are the perturbed indices (*V*’). Then, the sensitivity towards removing one or more features from the vulnerability analysis is calculated by:

(3) $$S=absolute\;value\left\{\frac{100}{V}\times \left(\frac{V}{N}-\frac{V^{'}}{n}\right)\right\}$$

where *S* is the sensitivity expressed in the form of index variation.

Having completed the estimation of aquifer vulnerability, the original or optimized *V* values are combined with a specific vulnerability index based on land uses or occupations (*L*), adapted from Antonakos and Lambrakis [4], to obtain the final score on groundwater contamination risk:

(4) Risk = *V* + 4 L

According to these authors, the most risky areas comprise the farmlands where fertilizers are used that can leach and contaminate the water table aquifer, while the less risky areas comprehend the forest spots where no potential sources of contamination are anticipated.

### Groundwater flow-contaminant transport module

The running of a groundwater flow-solute transport model such as the Processing Modflow (https://www.simcore.com) requires the availability of data on aquifer properties, namely hydraulic conductivity (*K*) and effective porosity (*n*_e_). The values of *K* and *n*_e_ can be estimated by the so-called method of Brutsaert [[24], [25], [26]], who related these properties to stream flow constants and catchment’s geometrical features. According to this method, in a plot of ln(Δ*Q*/Δ*t*) *versus*. ln(*Q*), where *Q* (m^3^·s^–1^) is the stream flow discharge rate and *t* (s) the corresponding time, the lower envelope to the scatter points is represented by two straight lines, one with a slope *b* = 1 representing the long-time flows (base flows), and the other with a slope *b* = 3 describing the short-time flows (inter flows). The *y*-values where the lines intercept ln(*Q*) = 0 (or *Q* = 1) are the stream flow constants *a*_1_ and *a*_3_, which are related to *K* and *n_e_* in Equations 5a,b:

(5a) $$K=0.57\sqrt{\frac{a_{1}}{a_{3}}}\left(\frac{A^{3}}{V_{z}^{2}L^{2}}\right)$$

(5b) $$n_{e}=\;\frac{1.98}{V_{z}\;\surd a_{1}a_{3}}$$

(5c) $$V_{z}=\;\gamma V$$

where *A*, *V* and *L* are the catchment’s geometric features area, volume and length of water channels, respectively, while *V*_z_ is the portion (γ) of *V* actually involved in groundwater flow defined as effective watershed volume. According to Santos et al. [27], γ = 0.25 for catchments larger than *A* = 10 km^2^. The method of Brutsaert has been successfully applied to spring and stream watersheds shaped on fractured rocks [28,29,30,27,31].

Although not essential to groundwater flow or solute transport modeling, the turnover time of groundwater in the aquifer can be estimated with the purpose to compare turnover times with stress periods of contaminant plume dispersion resulting from Processing Modflow runs. The turnover time (*t*, s) can be estimated through combination of stream flow constants and groundwater discharge (Q; m^–3^.s^–1^) as proposed in Pacheco [31]:

(6) $$t=\frac{1.98}{Q\;\surd a_{1}a_{3}}$$

The running of Processing Modflow starts with the creation of a new model and proceeds with generation of a grid (mesh) consisting of an array of nodes and associated finite-difference blocks (cells). Preparatory steps also include specification of aquifer types and boundary conditions, and the definition of hydrostratigraphic units represented by one or more model layers. The specification of boundary conditions is meant to identify no-flow, constant-head or active-head (time-dependent) cells. Having defined the model’s environment, it is necessary to populate the grid with topographic, hydrologic, aquifer property, and contaminant data. Processing Modflow requires the use of consistent units throughout the modeling process. The feeding of Processing Modflow with topographic data aims the definition of aquifer model geometry, namely the elevation of model layer limits (top and bottom). Usually, the top layer elevations are set to the altitudes of a local Digital Elevation Model (*H_top_*), while the bottom layer elevations (*H_bot_*) can be set to:

(7a) $$H_{bot}=H_{top}-b$$

with

(7b) $$b=\;\frac{V_{z}}{A}$$

Where *b* (m) is the average aquifer thickness estimated as ratio of effective watershed volume (Equation 5c) over catchment area. The required hydrologic and aquifer property data comprise estimates on hydraulic head (*h*), recharge (*R*), (horizontal) hydraulic conductivity (*K*) and effective porosity (*n*_e_). Hydraulic heads are calculated from topographic elevations (*H_top_*) and depths to the water table (*D*), which are the *D*_r_ values used in DRASTIC before their conversion into dimensionless ratings:

(8) $$h=\;H_{top}-D$$

As regards recharge, Processing Modflow can be sourced with the same estimates as used in the risk assessment module, while for *K* and *n*_e_ the software can use the outcomes from Equations 5a,b. In the case of *R* and *K*, the original units (mm·yr^–1^ and m·s^–1^, respectively) need prior conversion into the selected Processing Modflow units (e.g., m·day^–1^).

Processing Modflow is ready to run when temporal parameters and contaminant loads are finally specified. In this software, the simulation time is divided into the so-called stress periods, which are time intervals characterized by the constancy of external excitations or stresses. In the context of contaminant transport in rural catchments, the contaminant loads are defined where groundwater contamination derived from agriculture or livestock activities is expected, called “injection areas”. For the current workflow, contaminant loads were assumed to occur in arbitrary points distributed within the areas of high specific vulnerability determined on the basis of land use by the risk assessment module. Besides definition of injection areas and sites, it is necessary to set up the injected volume (*V*_i_; m^3^·day^–1^) and the contaminant’s maximum number of total moving particles. The injected volume was estimated by:

(9) $$V_{i}\;=R\times A$$

where *A* (m^2^) represents injection area and *R* the average recharge within that area. The modeled contaminant is nitrate, with the maximum number of total moving particles adjusted to the largest nitrate concentration observed in the studied area.

The simulations are based on the MODFLOW (for groundwater flow) and MT3D (for contaminant transport) tools of Processing Modflow software. The simulations involve the handling of advection and dispersion processes, but neglect any effect of chemical reaction. The parameters used in advection and dispersion packages are the default parameters (see Supplementary Material). The initial contaminant concentrations are set up to null values.

### Validation module

In this module, measured nitrate concentrations are compared with modeled nitrate concentrations along the catchment’s longest flow path. Firstly, the measured and modeled (for each stress period) are digitally sampled along the longest flow line vertices. The sampled values are saved in table format (see Supplementary Material) and opened in Microsoft Excel. The sampled values comprise the distance from flow line origin and the nitrate concentration at the flow line vertex. Subsequently, nitrate concentrations evaluated at the vertices are plotted as function of distance. This plot is called a cross profile. Measured and modeled concentrations are represented in the same diagram with the purpose of identifying the best match between real and predicted profiles. The process is repeated if nitrate concentrations have been measured at different times (e.g. winter, summer) in different flow components (e.g., interflow / return flow or base flow). For the best matches, a coefficient of determination is estimated between measured and modeled concentrations to be used as goodness of fit measure.

### Insights on model implementation

The base data required to implement the workflow is demanding and diverse. Usually, it needs to be compiled from various sources, as demonstrated in Table 1. Module 1 uses a great deal of geographical data. Most intrinsic / specific vulnerability parameters are spatially represented by polygon shapefiles often related to qualitative parameters (e.g., parameter A - aquifer material), points linked to numerical parameters (e.g., parameter D – depth to the water table assessed in dug wells), or raster files also linked to numerical parameters (e.g. parameter T – topography). Lines are less frequently used unless, for example, fractures are combined with lithologic types to represent the A parameter. The attribute table of polygon shapefiles (e.g., lithology) needs to contain a column where the associated parameter (i.e., A) is rated. The raster map displaying the categorical parameter in space is obtained through polygon to raster conversion within the ArcMap software, using the aforementioned column as conversion field. The handling of point shapefiles is different. In general, the original variable (e.g. recharge) is first interpolated across the studied area and then recast as ratings (i.e., R) in keeping with the DRASTIC model reclassification tables. Interpolation and reclassification tools are available from the ArcMap toolbox. Digital elevation models in raster format are usually the source data for parameter T. Before converting elevation data into T ratings the user needs to use the slope tool of ArcMap to obtain slope values in percent rise.

The stage of sensitivity analysis is carried out in Microsoft Excel. Firstly, the raster maps displaying the DRASTIC parameters are digitally sampled (Sample tool from the Spatial Analyst > Extraction toolbox) using a mesh of regularly distributed points as input location feature. Secondly, the result is exported as table and subsequently imported into Excel. Finally, Equations 2 and 3 are implemented in this software as can be consulted in the Supplementary Material.

The DRASTIC (Equation 1) and risk (Equation 4) maps are obtained through the map algebra tool of ArcMap.

Module 2a combines the use of ArcHydro and Excel. The ArcHydro tools are embedded in ArcMap and are used to draw water lines and catchment boundaries as well as to evaluate their geometric parameters (*A*, *V*, *L*). The scores of *A*, *V* and *L* are then used in Equations 5a-c to estimate *K* and n_e_. However, before completing this task, stream flow constants *a*_1_ and *a*_3_ need to be estimated from the ln(Δ*Q*/Δ*t*) *versus*. ln(*Q*) diagrams. The Supplementary Material contains indication on how the lower envelopes to the scatter points are used to obtain these values. Having estimated a_1_ and a_3_, the turnover time of groundwater is calculated straightforwardly by combining these values with average spring discharges in Equation 6.

Implementation of Module 2b requires the export of a number of parameters (e.g., *H*_top_, *H*_bot_, *b*, *h*; Equations 7 and 8) in ASCII format to the Processing Modflow software, using the raster to ASCII conversion tool of ArcMap. The ASCII files are headed by the number of rows and columns defining the modeling grid, followed in the succeeding lines by the parameter values at the grid nodes (please see Supplementary Material).

Implementation of Module 3 requires no further insights besides the ones presented above.
